# Validation of the Mobile App–Recorded Circadian Rhythm by a Digital Footprint

**DOI:** 10.2196/13421

**Published:** 2019-05-16

**Authors:** Yu-Hsuan Lin, Bo-Yu Wong, Yuan-Chien Pan, Yu-Chuan Chiu, Yang-Han Lee

**Affiliations:** 1 Institute of Population Health Sciences National Health Research Institutes Miaoli Taiwan; 2 Department of Psychiatry National Taiwan University Hospital Taipei Taiwan; 3 Department of Psychiatry College of Medicine National Taiwan University Taipei Taiwan; 4 Institute of Health Behaviors and Community Sciences College of Public Health National Taiwan University Taipei Taiwan; 5 Department of Psychology National Taiwan University Taipei Taiwan; 6 Department of Psychiatry MacKay Memorial Hospital Taipei Taiwan; 7 Department and Graduate School of Electrical Engineering Tamkang University New Taipei City Taiwan

**Keywords:** circadian rhythm, sleep, smartphone, mobile applications

## Abstract

**Background:**

Modern smartphone use is pervasive and could be an accessible method of evaluating the circadian rhythm and social jet lag via a mobile app.

**Objective:**

This study aimed to validate the app-recorded sleep time with daily self-reports by examining the consistency of total sleep time (TST), as well as the timing of sleep onset and wake time, and to validate the app-recorded circadian rhythm with the corresponding 30-day self-reported midpoint of sleep and the consistency of social jetlag.

**Methods:**

The mobile app, Rhythm, recorded parameters and these parameters were hypothesized to be used to infer a relative long-term pattern of the circadian rhythm. In total, 28 volunteers downloaded the app, and 30 days of automatically recorded data along with self-reported sleep measures were collected.

**Results:**

No significant difference was noted between app-recorded and self-reported midpoint of sleep time and between app-recorded and self-reported social jetlag. The overall correlation coefficient of app-recorded and self-reported midpoint of sleep time was .87.

**Conclusions:**

The circadian rhythm for 1 month, daily TST, and timing of sleep onset could be automatically calculated by the app and algorithm.

## Introduction

### Background

Human beings, like other animals and plants, have a biological clock that helps to prepare their physiology for the fluctuations of the day. This regular adaptation is referred to as the circadian rhythm. Since the 1970s, scientists have investigated the molecular mechanisms controlling the circadian rhythm [1-7]. Chronic circadian dysregulation has recently been implicated in the increased risk of cancer, neurodegenerative disorders, metabolic disorders, and inflammation [8]. Circadian disruption has also been associated with several psychiatric disorders, such as bipolar disorder, major depression, and schizophrenia. However, although research has led to a better understanding of the biological basis of the circadian rhythm, most clinical studies are conducted either in the artificial settings of a laboratory (eg, polysomnography), which are not scalable for administration to a large population [9], or through subjective self-report questionnaires with the value reduced by biases [10-12].

Nowadays, human circadian rhythms could be observed from their digital footprint. Digital footprint refers to data rising from day-to-day interactions with newer technologies such as smartphones [13]. Real-time and passively collected data can provide a long-term recording of the circadian rhythm in a naturalistic setting and contribute toward self-awareness or clinical applications, such as sleep diary and social jetlag estimation. In addition, smartphone ownership has shown to not be affected by socioeconomic status [14]. Given the convenience of smartphones, health-related mobile apps might serve as a *digital lifeline*, particularly in rural and low-income regions, helping mental health care professionals with medical intervention and behavioral modification [15]. The widespread use and deep reach of smartphones in modern life motivate the use of smartphones to measure behaviors in an affordable, reliable, and unobtrusive way.

### Objective

This pilot study had proposed that the longest nonusage episodes during night-time could represent actual sleep time [16]. A previous study also preliminarily validated that the consistency between app-recorded and self-reported sleep time was 83.0%. However, this validation was based on 14-day app-recorded data with 1 self-reported weeknight and weekend night sleep time. The current version of the app, *Rhythm*, with 2 major algorithm revisions is hypothesized to improve the consistency of app-recorded and self-reported sleep time. In addition, the app-recorded sleep indicators, especially as midpoint of sleep time, can be used to infer a relative long-term pattern of the circadian rhythm. The specific aims of this study were (1) to validate the app-recorded sleep time with daily self-reports by examining the consistency of total sleep time (TST), as well as the timing of sleep onset and wake time, and (2) to validate the app-recorded circadian rhythm with the corresponding 30-day self-reported midpoint of sleep and the consistency of social jetlag.

## Methods

### Participants and Procedure

A total of 28 college students (13 men, mean age 20.8 (SD 1.6) years, range 17 to 23) were recruited. The sleep time data were collected by the mobile app, *Rhythm*, from February to June 2018. After informed consent, the participants were asked to install Rhythm for at least 30 days. Data collected on the first day and the last day were excluded owing to the incomplete nature of data on those dates. The app-recorded sleep data of the first 30 days of each participant were selected to be analyzed. The 30-day data consisted of about 16.9 days weekday data and 13.1 days weekend data.

This app automatically estimated sleep onset and wake time via an algorithm daily, and participants received a notification from this app to show their sleep onset and wake time last night at 21:00 every night. Then participants were asked to adjust the sleep onset and wake time as their self-reported sleep time. If the difference of sleep time between self-reported and app-recorded was greater than 2 hours, the researchers would confirm the self-reported sleep time with participants via a phone call. The participants were blind to the purpose of the confirmation process. The study was approved by the Institutional Review Board of National Health Research Institutes. All clinical investigations were conducted according to the principles expressed in the Declaration of Helsinki.

### Measures

#### Designing the App, Rhythm

The app, *Rhythm*, automatically recorded smartphone behaviors, especially the notifications and screen-on and screen-off timing. This app collects data in the background without interrupting smartphone operation or impacting battery life (less than 1%) [16-18]. The app saves all recorded behavior data in a log file and routinely uploads to the database every midnight (01:00) and the following noon (12:00). The sleep indicators were calculated, and the server would send a notification with their sleep onset, wake time, and TST at 21:00. We choose 21:00 as the time to send the notification because these sleep indicators could not be calculated in real time on their smartphones. In addition, participants might easily ignore these notifications during their working hours.

**Figure 1:**
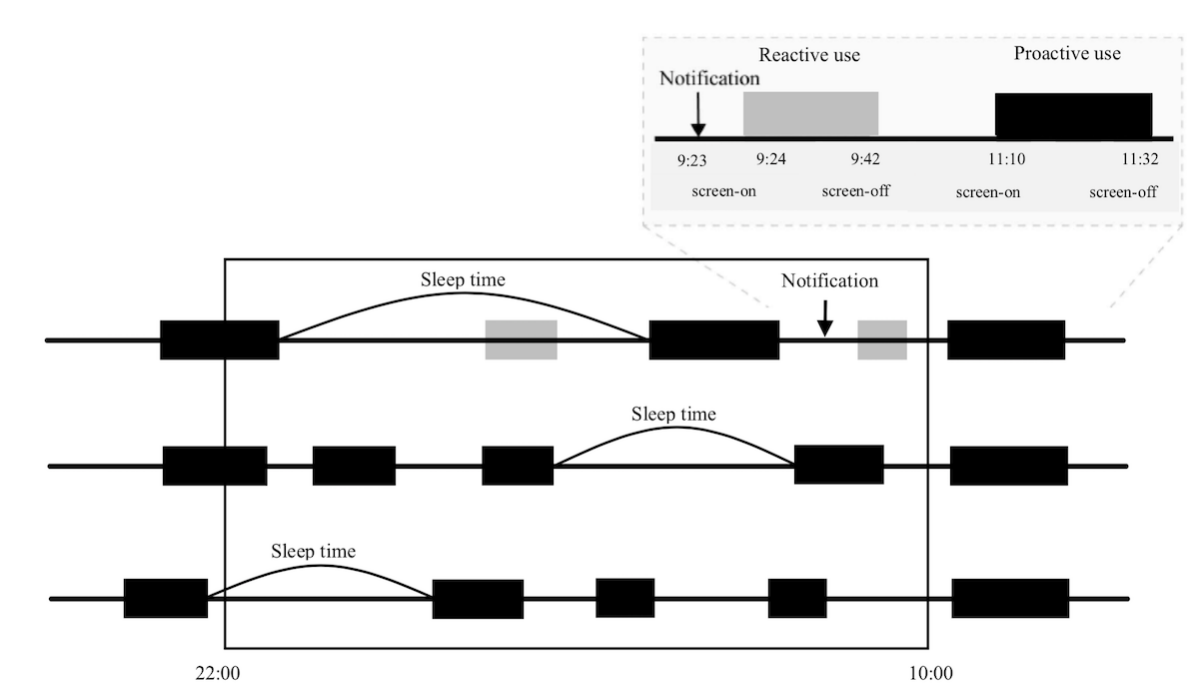
Definitions of reactive use and proactive use and the rules of identifying sleep time via smartphone use data.

Smartphone use from screen-on to the successive screen-off was defined as one episode of use. This app calculated the daily total duration of smartphone usage episodes. The usage episode with no notification within 1 min before screen-on was classified into proactive use. The upper right box of Figure 1 shows a sample of reactive use and proactive use. Only proactive usage episodes were included in the calculation of sleep time. To avoid a high number of frequent notifications confounding proactive use as reactive use, which has a notification within 1 min before screen-on, all notifications from the app which presented more than 500 times per day were excluded. In contrast to these proactive usage episodes, the event from screen-off to screen-on was defined as the nonusage episode.

Figure 1 shows 3 examples to demonstrate the algorithm of identifying sleep time via smartphone use data. For the first example, the maximal nonusage episode between 22:00 and the following 10:00 is defined as *sleep time*. The other 2 examples demonstrated an additional algorithm to identify the sleep onset or wake time not located between 22:00 and 10:00. First, the dummy screen-off (sleep onset) at 22:00 and screen-on (wake time) at 10:00 were labeled to the nonusage episodes with the screen-off before 22:00 and screen-on after 10:00. Second, if the maximal nonusage episode between 22:00 and the following 10:00 was labeled a dummy screen-off at 22:00 or screen-on at 10:00, the original screen-off before 22:00 or screen-on after 10:00 was resumed as the timing of sleep onset and wake time, respectively. Finally, the sleep onset, wake time, midpoint of sleep, and TST could be identified.

### Validation of the App-Recorded Sleep Indicators and Circadian Rhythm

#### Validation of the App-Recorded Sleep Indicators

**Figure 2:**
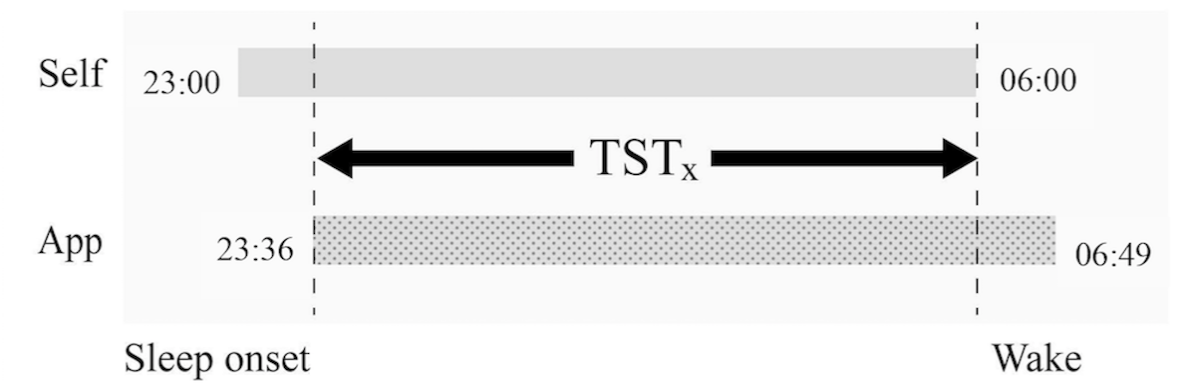
Definition of sleep onset time, midpoint of sleep, and wake time. TST: total sleep time.

A paired *t* test was used to examine the differences between app-recorded and self-reported indicators, namely sleep onset, wake time, midpoint of sleep time, and TST. Figure 2 shows the consistency of the app-recorded and self-reported sleep time by an overlap ratio [19], and the overlap ratio can mathematically be expressed as follows:

Overlap Ratio = Overlap (TST_x_) / [(TST_app_ + TST_self_) / 2] × 100%

The app-recorded and self-reported TST can be calculated as TST_app_ and TST_self_. The overlapping TST between app-recorded and self-reported TST is defined as TST_x_. In Figure 2, the overlap ratio for the example is:

(23:36 ~ 06:00) / {[(23:00 ~ 06:00) + (23:36 ~ 06:49)] / 2} = 90.0%

In addition, a paired *t* test was used to compare these overlap ratios on weeknights and weekend nights.

#### Validation of the Circadian Rhythm

The Pearson correlation coefficient of app-recorded and self-reported midpoint of sleep time within a participant’s 30-day data was calculated along with the average midpoint of sleep differences between weeknights and weekend nights, and these differences are social jetlag [20,21]. Then, a paired *t* test was used to examine the difference between app-recorded and self-reported social jetlag.

All statistical assessments were 2-tailed, and *P*<.05 was considered to be statistically significant. Statistical analyses were per­formed using SPSS version 18.0 software (SPSS Inc).

## Results

The average total daily smartphone use duration of the participants was 5.73 (SD 3.42) hours. Table 1 shows that there is no significant difference between app-recorded and self-reported midpoint of sleep time. App-recorded sleep onset had a 242.9-second delay from self-reported onset with a borderline *P* value (.053). App-recorded wake time had a significant 623.7-second advance to self-reported wake time (*P*=.001). App-recorded TST had a significantly shorter 866.6 seconds than self-reported time (*P*<.001). However, the overlap ratio of app-recorded and self-reported was 90.4%. There was no significant difference of overlap ratio between weeknights and weekend nights (*P*=.213).

**Figure 3:**
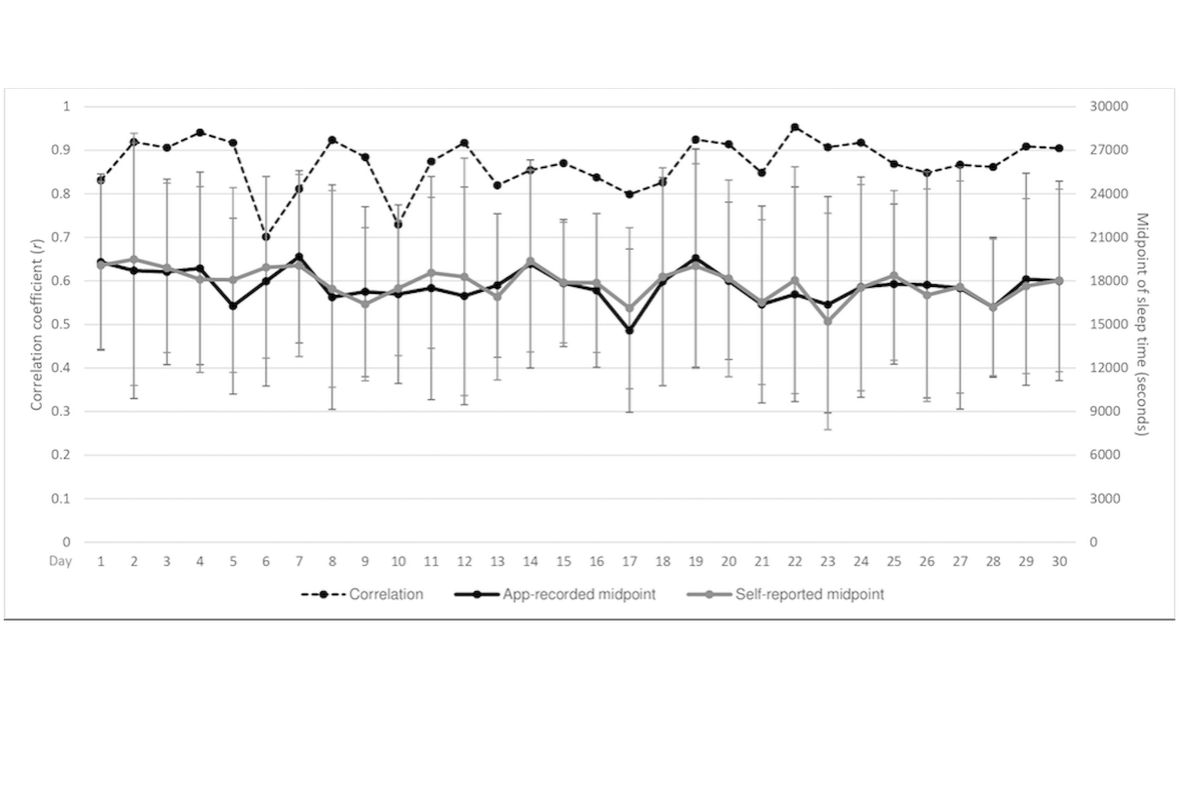
Circadian rhythm fluctuations for app-recorded and self-reported midpoint of sleep time with 1 SD error bar.

Figure 3 shows 28 participants’ average app-recorded and self-reported midpoint of sleep time and the correlation coefficients from Day 1 to Day 30. These daily correlation coefficients ranged from .70 to .95, and the overall coefficient between app-recorded and self-reported midpoint of sleep time was .87 (*N*=840). There is no significant difference (*P*=.140) between app-recorded (34.4 [SD 52.5] min) and self-reported social jetlag (27.0 [SD 49.8] min).

**Table 1:** The consistency between app-recorded and self-reported sleep indicator.

Sleep indicators	Elapsed time since midnight (seconds)					
	App-recorded		Self-reported		*t*_27_	*P* value
	Average time	Mean (SD)	Average time	Mean (SD)		
Sleep onset	01:26:43	5202.53 (7093.71)	01:22:40	4959.64 (6662.52)	1.94	.053
Wake	08:21:44	30104.20 (9037.06)	08:32:08	30727.93 (8417.06)	−3.35	.001^a^
Midpoint	04:54:13	17653.27 (6707.71)	04:57:24	17843.74 (6362.29)	−1.64	.103
Total sleep time	06:55:02	24901.67 (9165.46)	07:09:28	25768.30 (8279.86)	−4.02	<.001^b^

^a^*P*<.05.

^b^*P*<.001.

## Discussion

### Principal Findings

After a review of the literature, this is the first study to validate an innovative approach to automatically record 1-month circadian rhythm and sleep time by a mobile app. The high correlation (*r*=.87) between app-recorded and self-reported circadian rhythm was validated by 28 participants and their 30-day sleep-wake cycles with a total of 840 pairs of app-recorded data and self-reports. Collecting data passively from a person’s smartphone (app) may be more informative than self-reports. This data collection method can provide continuous monitoring over longitudinal periods. In addition, the value of self-reports might be reduced by biases [13], whereas data collection by this automatically operated app is less prone to biases, as the process of collecting data from everyday interactions with technology is unlikely to induce reactivity. In addition, of note is that the temporal resolution of self-reported sleep time is usually 1 hour [22]. Compared with the temporal resolution of 1 second in this study, these app-generated parameters increased the temporal resolution 3600-fold compared with the resolution in conventional epidemiology surveys.

### Literature Review/Related Work

There have been several mobile apps on the market to measure sleep automatically via smartphone sensors. Best Effort Sleep [23] uses a sensor-based inference algorithm that combines smartphone usage patterns along with environmental cues such as light and ambient sound to infer a user’s sleep duration. Similarly, Toss ‘N’ Turn [24] also collects sound, light, movement, screen state, app usage, and battery status to classify sleep state and quality. The systems, iSleep [25] and wakeNsmile [26], use a built-in phone microphone to detect body movement and sounds such as cough and snoring to predict sleep phases. However, such apps typically assess sleep time or sleep phases but these mobile apps do not take the circadian rhythm into consideration.

These mobile sensing-based algorithms with less power consumption would advantage from delineating the circadian rhythm from a long consecutive sleep recording. Only a couple of mobile apps compute the sleep time and circadian rhythm solely based on smartphone usage patterns. The pilot study we performed identified proactive smartphone screen-on and screen-off patterns to estimate sleep time and achieved 83% accuracy [19]. UbiComp [27] similarly showed that smartphone usage patterns were able to detect sleep duration as well as symptoms of sleep deprivation. Although these mobile sensing-based apps were validated to assess sleep time, this is the first study to validate both sleep time and circadian rhythm for 30 days with corresponding day-by-day self-reports.

### Strengths

Technically, 2 additional criteria were added to improve the algorithm of sleep time estimation with the consistency of 90.4% in this study. First, the extended tracing of sleep onset before 22:00 and wake time after 10:00 could identify the relative irregular circadian rhythm compared with the other methods that limited sleep time between 22:00 and 10:00. Second, the exclusion of frequent notifications elaborated the differentiation from proactive and reactive use and delineated the sleep time more precisely. These 2 improvements in the present algorithm promoted the accuracy of TST estimation from 83.0% [19] to 90.4%. Owing to the extended tracing of the circadian rhythm and much precise sleep time identification, the correlation coefficient of app-recorded and self-reported 1-month circadian rhythm was .87. In addition, the app-recorded and self-reported social jetlag was similar, without any significant difference. These findings showed that using a smartphone to record passive data, the timing of screen-on and screen-off, and notifications could automatically calculate the sleep time and circadian rhythm for 1 month.

Despite no significant difference between the app-recorded and self-reported sleep time midpoints, the app-recorded wake time was significantly earlier than self-reports by 10.4 min (623.7 seconds) and app-recorded sleep onset was longer than self-reports by 4.0 min (242.9 seconds) with a borderline *P* value (.053). The later sleep and earlier wake timing of app records counteracted the differences in midpoints of sleep time, the indicators of circadian rhythm. Therefore, the 1-month circadian rhythm based on the midpoint of sleep correlation of app records and self-reports reached .87 in this study. The study results revealed that it is feasible to monitor the circadian rhythm automatically by this app that we developed. However, the app-recorded TST might be 14.4 min (866.6 seconds) shorter than the self-reported time despite the consistency of sleep time being 90.4%.

The differences of sleep and wake timing between app records and self-reports might have resulted from some habitual bedtime behaviors, such as bedtime smartphone use before sleep and lying in bed after waking. In addition, distorted time perception [18] also play an important role in these timing differences, especially in participants with average daily smartphone use of 5.84 (SD 2.92) hour/day in this study. The participants might use the smartphone at waking up and might have stayed in bed for an average 10.4 min to be aware that they actually woke up. In this condition, the app could estimate time in bed by self-reported TST and sleep time by app-recorded TST. Therefore, the sleep efficiency, defined as the ratio of sleep to time in bed, could be estimated by TST_app_/TST_self_. Although the gold standard method to define the sleep and wake timing is polysomnography, this app-recorded sleep-wake cycle in a naturalistic environment has provided a more cost-efficient and convenient way to continuously delineate the circadian rhythm.

### Limitations

There are several methodological limitations that should be noted when interpreting the study’s findings. First, the study utilized a selected sample with excessive smartphone use (average daily smartphone use duration: 5.84 (SD 2.92) hour/day) and late chronotype (average sleep onset at 01:26:43 and TST: 6.92 hours). A previous study had demonstrated the association between excessive internet use and late chronotype [10]. In addition, both excessive smartphone use and late chronotype might limit the ability to generalize these findings because this algorithm to estimate sleep patterns depended on participants’ smartphone events. Using smartphone use data combined with an activity wristband that gathers the whole day’s activities and physiological indicators could improve the reliability of computing the circadian rhythm for participants owning such devices. Second, the app was based on the Android operating system. Various versions applicable to other operating systems such as iOS and Windows should be developed in the future. In addition, the app failed to record notifications in 1 smartphone brand. However, it is essential to calculate the sleep time by screen-on, screen-off, and notifications. Third, this algorithm to determine sleep time could not account for sleep interruptions with proactive smartphone use or shift workers’ daytime sleep. This algorithm should be adjusted and validated for patients with sleep disturbance and shift workers. Finally, although these app-recorded sleep indicators were validated by participants’ daily self-reports, it would be better to use the current gold standard to assess circadian rhythm, actigraphy, to validate the app-recorded sleep time in a future study.

### Conclusions

In conclusion, this study validated the algorithm of sleep estimation and circadian rhythm by using the app, *Rhythm*, that can collect passive data from naturalistic settings. The circadian rhythm for 1 month, daily TST, and timing of sleep onset could be automatically calculated by the app.

## Abbreviations

TST: total sleep time
